# Missense mutation of *MAL* causes a rare leukodystrophy similar to Pelizaeus-Merzbacher disease

**DOI:** 10.1038/s41431-022-01050-9

**Published:** 2022-02-25

**Authors:** Marilena Elpidorou, James A. Poulter, Katarzyna Szymanska, Wia Baron, Katrin Junger, Karsten Boldt, Marius Ueffing, Lydia Green, John H. Livingston, Eammon G. Sheridan, Colin A. Johnson

**Affiliations:** 1 Division of Molecular Medicine, Leeds Institute of Medical Research, University of Leeds, Leeds, UK; 2 Department of Biomedical Sciences of Cells & Systems, Section of Molecular Neurobiology, University of Groningen, University Medical Center Groningen, Groningen, The Netherlands; 3 Centre for Ophthalmology, Institute for Ophthalmic Research, University Hospital of Tübingen, Tübingen, Germany; 4 Department of Paediatric Neurology, Leeds Teaching Hospitals NHS Trust, Leeds, UK; 5 Department of Clinical Genetics, Leeds Teaching Hospitals Trust, Leeds, UK

**Keywords:** Genetics research, Medical genetics, Sequencing

## Abstract

Leukodystrophies are a heterogenous group of genetic disorders, characterised by abnormal development of cerebral white matter. Pelizaeus-Merzbacher disease is caused by mutations in *PLP1*, encoding major myelin-resident protein required for myelin sheath assembly. We report a missense variant p.(Ala109Asp) in *MAL* as causative for a rare, hypomyelinating leukodystrophy similar to Pelizaeus-Merzbacher disease. *MAL* encodes a membrane proteolipid that directly interacts with PLP1, ensuring correct distribution during myelin assembly. In contrast to wild-type MAL, mutant MAL was retained in the endoplasmic reticulum but was released following treatment with 4-phenylbutyrate. Proximity-dependent identification of wild-type MAL interactants implicated post-Golgi vesicle-mediated protein transport and protein localisation to membranes, whereas mutant MAL interactants suggested unfolded protein responses. Our results suggest that mislocalisation of MAL affects PLP1 distribution, consistent with known pathomechanisms for hypomyelinating leukodystrophies.

## Introduction

Genetic forms of leukodystrophy are a heterogenous group of congenital hypomyelinating or demyelinating disorders, resulting from deficient or abnormal myelin deposition within the CNS. Leukodystrophies have varied clinical and radiological phenotypes, and the largest group are hypomyelinating leukodystrophies that have significant and permanent deficit of myelin. *PLP1* mutations are the most common, causative for Pelizaeus-Merzbacher disease [1]. *PLP1* encodes proteolipid protein 1, the major myelin-resident protein required for myelin sheath assembly in nerve fibres [2]. Correctly-timed trafficking of PLP1 is fundamental to myelin biogenesis during development [3].

*MAL* (myelin and lymphocyte protein) encodes a membrane proteolipid with four transmembrane domains mainly localised in compact myelin that is highly expressed in pre-mature Schwann cells, oligodendrocytes and mature Schwann cells [4]. MAL directly interacts with PLP1, redirecting PLP1 transport towards myelin membranes [2]. MAL also mediates vesicular trafficking including direct apical transport from the Golgi apparatus (essential for myelination by oligodendrocytes) and transcytosis to basolateral membranes [5].

Here, we report the identification and characterisation of a missense variant in *MAL* as causative for a rare, hypomyelinating leukodystrophy. As previously observed for *PLP1* mutations, mutated MAL protein was retained in the endoplasmic reticulum (ER) [6, 7] and was unable to bind PLP1. Our results suggest that mislocalisation of MAL affects the distribution of PLP1, consistent with known pathomechanisms for Pelizaeus-Merzbacher disease.

## Materials and methods

### Patients

Family members were recruited with informed consent and under ethical approval from South Yorkshire Research Ethics Committee (REC ref. no. 11/H1310/1). Genomic DNA was obtained from blood samples as described previously [8].

### Whole exome sequencing and bioinformatics analysis

**Fig. 1:**
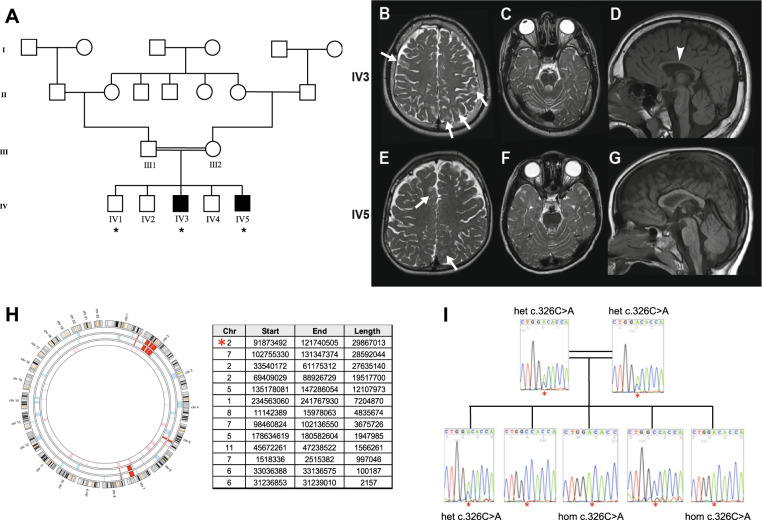
Clinical ascertainment and genetic analysis. **A** Pedigree of the family under study, summarising four generations and the consanguineous union of the parents of the two affected individuals (IV3 and IV5). Individuals recruited to the study are indicated by Roman numerals, and individuals whose DNA samples have been used for WES are marked with a star (*). **B**–**G** MR images of individual IV3 (**B**–**D**) aged 14 years, and his brother IV5 (**E**–**G**) aged 6 years. T2 axial images (**B**, **C** & **E**, **F**) demonstrating lack of hemispheric myelin visible through T2 hyperintensity (brightness indicated by white arrows). Prominence of cerebellar folia and vermian hypoplasia (**C** & **F**) indicates cerebellar volume loss. T1 sagittal images (**D** & **G**) demonstrate a thin corpus callosum (**D**, indicated by white arrowhead) and mild upper vermian volume loss. **H** Ideogram illustrating the shared homozygous regions of the two affected individuals in red, and a table summarising the significant homozygous regions in chromosome order. The largest homozygous region on chromosome 2 that contains *MAL* is marked by the red asterisk (*). **I** Segregation analysis of the *MAL* c.326 C > A p.(Ala109Asp) variant (indicated by red asterisk, *) within the family confirmed by Sanger sequencing. het heterozygous, ho homozygous.

Whole exome sequencing (WES) was performed using genomic DNA from two affected and one unaffected sibling (indicted by *, Fig. 1A) in the family. DNA was processed using the Agilent ‘SureSelect QXT’ Target Enrichment kit (Agilent Technologies). DNA libraries were sequenced on an Illumina HiSeq 3000 using a 150 bp paired-end protocol. Bioinformatics analysis was done using standard pipelines (Supplementary Methods A).

### Molecular biology and other methods

PCR, Sanger sequencing, cDNA insert cloning and site-directed mutagenesis are summarised in Supplementary Methods B–C. Cell culture, transfection, western blotting and immunoprecipitation methodologies were performed as described previously [8], summarised in Supplementary Methods D–F. BioID2 and mass spectroscopy analysis is outlined in Supplementary Methods G. Confocal imaging and live cell imaging are detailed in Supplementary Methods H. Statistical analysis is summarised in Supplementary Methods I.

## Results

### Clinical ascertainment

Two affected male siblings presented with developmental delay and nystagmus in infancy, followed by significant learning disabilities and progressive motor deterioration within the first decade. The older sibling also had epilepsy. Both patients were described to be increasingly unsteady with falls. Examination of the younger sibling aged 8 demonstrated increased lower limb tone, brisk deep tendon reflexes and new onset ataxia. He had intention tremor and mild dysmetria. MR imaging demonstrated cerebellar volume loss with patchy dysmyelination in subcortical areas in T2 sequences (Fig. 1A–G) that is different from Pelizeaus-Merzbacher patients who usually have a diffuse hypomyelination, mainly in periventricular regions and semi-ovale centres. Sequencing of *PLP1* did not reveal pathogenic mutations and an MLPA assay excluded *PLP1* duplication. No further mutations were identified in a diagnostic panel for white matter-associated genes (Supplemental Data A). The parents of the affected siblings were first cousins of Pakistani origin (Fig. 1A).

### Whole exome sequencing and in silico modelling

**Fig. 2:**
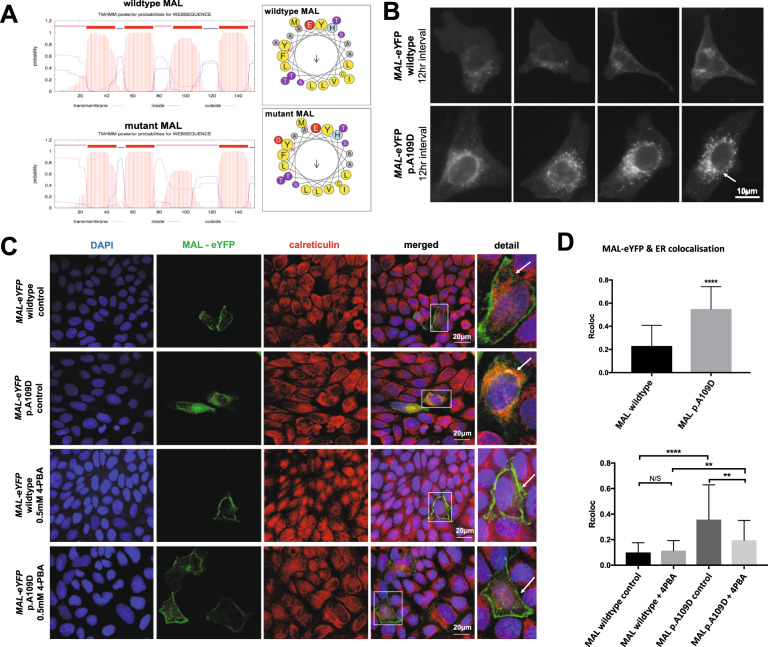
The MAL p.(Ala109Asp) mutation causes protein aggregation in the ER. **A** Computational prediction of the effect of the p.(Ala109Asp) variant on the transmembrane helix organisation of the MAL protein using the TMHMM Transmembrane Helix prediction tool (left). Top image indicates that each of the transmembrane helices (red) in the wildtype MAL protein have the predicted organisation of a tetraspanin-like protein, but the third transmembrane domain in p.(Ala109Asp) mutant MAL is predicted to no longer span the membrane as a result of the hydrophilic amino acid substitution. HeliQuest validated this change (right) showing that the hydrophobic phase of the wildtype helix (LFYAAAM; residues coloured yellow) is compromised by the mutation (**D**; red). **B** Live cell imaging of MAL wildtype and MAL p.(Ala109Asp) mutant C-terminal eYFP-tagged proteins, visualised using a Nikon BioStation system for a total of 24 h. The wildtype MAL protein localised at the cell membrane and some perinuclear regions, whereas mutant MAL protein formed prominent aggregates in perinuclear regions of the ER (arrow). Scale bar = 10 μm. **C** Immunofluorescence staining and confocal microscopy imaging of wildtype and mutant MAL eYFP-tagged protein overexpression in MDCK cells, following 4-phenylbutyrate (4-PBA) treatment, calreticulin (red) and DAPI (blue). Co-localisation of mutant MAL-eYFP and calreticulin (detail visible in magnified insets; indicated by white boxes) in ER aggregate (arrows) is reduced following 4-PBA treatment. IF experiments were performed in three independent biological replicates, each of three technical replicates each, with analysis of a total of 120 cells. Scale bar = 20 μm. **D** Top graph: co-localisation analysis of MAL protein and the ER marker calreticulin, using Fiji to determine Pearson R^2^ “Rcoloc” correlation values for each transfected cell. There is a significant increase in ER colocalization for mutant MAL protein compared to the wildtype. Analysis included a total of 100 cells, from three independent biological replicates. Statistical analysis of a pairwise comparison was performed using a two-tailed Student *t* test (****p < 0.0001). Error bars indicate s.e.m. Bottom graph: analysis of MAL protein and ER colocalization, for control cells or following 4-PBA treatment. Rcoloc values for each cell analysed indicate a significant difference in ER colocalization for the treated cells expressing mutant MAL compared to the untreated control cells expressing mutant MAL. Statistical analysis of pair-wise comparisons was performed by a two-tailed Student *t* test (ns not significant; *p < 0.05, **p < 0.01, ***p < 0.001, ****p < 0.0001). Error bars indicate s.e.m.

Whole exome sequencing was performed on the two affected boys and an unaffected sibling. Variants incompatible with autosomal recessive or X-linked inheritance were filtered out. Autozygosity mapping allowed prioritisation of variants within regions identical-by-descent (Fig. 1H), and three variants were identified that passed filtering criteria (Supplementary Data B). One variant did not segregate in the family and a second was in a gene, *ZNHIT1*, that was an unlikely functional candidate (Supplementary Fig. 1). A homozygous variant in MAL, p.(Ala109Asp), segregated in the family (Fig. 1I) was predicted to be pathogenic by Condel, Polyphen2 and SIFT, had a CADD score of 34, and was absent from dbSNP151 and gnomAD (v2.1.1). In silico modelling MAL p.(Ala109Asp) predicted alpha-helical secondary structure was lost (HeliQuest) preventing insertion of the third transmembrane domain into a membrane (Transmembrane Helix Prediction TMHMM Server v.2.0; Fig. 2A).

### MAL p.(Ala109Asp) forms aggregates in the endoplasmic reticulum that are decreased by the chemical chaperone 4-phenylbutyrate

Wild-type MAL-eYFP fusion protein localised to perinuclear and basolateral lineate localisations at the plasma membrane in polarised MDCK cells (Fig. 2B), whereas mutant MAL protein co-localised in aggregates containing calreticulin, a marker of the ER (Fig. 2C–D). ER protein aggregates form when newly-synthesised proteins fail to fold correctly, leading to ER stress and activation of unfolded protein responses (UPR) [9, 10]. Treatment with 4-phenylbutyrate, a chemical chaperone, significantly decreased co-localisation of mutant MAL p.(Ala109Asp) protein with calreticulin (Fig. 2C–D) suggesting that it corrected protein folding.

### Missense variant p.(Ala109Asp) abrogates the interaction with PLP1 and correct distribution to basolateral membranes

**Fig. 3:**
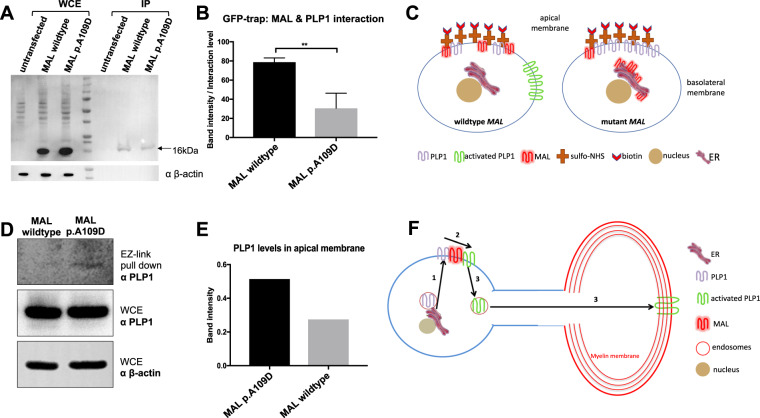
The MAL p.(Ala109Asp) mutation abrogates the interaction between MAL and PLP1. **A** Western blot analysis of immunoprecipitations to assess interactions between PLP1-GFP protein and either wildtype or mutant MAL C-terminal V5-tagged protein. Pull-down was performed with anti-GFP beads and westerns blots resolved with anti-V5 antibody. The arrow indicates MAL protein (expected size 16 kDa). **B** Quantification of wildtype or mutant MAL and PLP1 interactions from three independent biological replicates. Statistical analysis of pair-wise comparisons was performed by a two-tailed Student *t* test (**p < 0.01). Error bars indicate s.e.m. **C** Schematic of the EZ-Link-Sulfo-NHS-Biotin biotinylation assay, in order to assess the localisation of PLP1 for cells expressing either wildtype or mutant MAL. On the left, PLP1 correctly localises to the basolateral membrane in cells expressing wildtype MAL but remains in the apical membrane in the mutant MAL model. **D** Western blot results from the biotinylation assay showing a reduction in PLP1 levels from the apical membrane for cells expressing wildtype MAL compared to mutant MAL model. Total input PLP1 levels are determined by anti-PLP1 western blotting of whole cell extracts (WCE) and β-actin is a loading control. **E** Quantification of the western blot results using Image Lab. Anti-PLP1 band intensity from the pull-down was normalised to anti-PLP1 bands in WCE, corrected for overall loading using β-actin levels. Data presented is for two independent biological replicates. **F** Proposed mechanism of the role of MAL as a regulator of intracellular PLP1 trafficking. Nascent PLP1 is trafficked from the ER to the apical cell membrane (arrow 1). MAL and PLP1 interact at the apical membrane of myelinating oligodentrocytes (arrow 2), where MAL redirects PLP1 localisation to the basolateral membrane (arrow 3) during myelin formation.

In oligodendrocytes, MAL acts as a regulator of PLP1 transcytosis during myelin formation [2, 3]. Quantification of immunoprecipitation assays following co-expression of MAL-V5 and PLP1-GFP-tagged proteins revealed a 56% loss of interaction between mutant MAL and PLP1 (Fig. 3A–B). Next, we used the EZ-Link-Sulfo-NHS-Biotin assay to biotinylate lysine residues in apical cell surface proteins (Fig. 3C). Apically mislocalized PLP1 was detected in cells expressing mutant MAL whereas wild-type protein was hardly detectable, suggesting that correct redirection of PLP1 from apical to basolateral membranes only occurs in cells expressing wildtype MAL.

### Proximity-dependent interactants of wild-type and mutant MAL

Expression of wild-type and mutant MAL-BirA2 (Supplementary Fig. 2) for ‘BioID’ proximity-dependent biotinylation identified candidate interacting proteins required for PLP1 transcytosis. Lysates were pulled-down with streptactin beads, trypsinised and then analysed using mass spectroscopy (Supplementary Data C). MAL and PLP1 proteins had limited trypsin cleavage sites, and their presence in the pull-downs was confirmed using western blotting (Supplementary Fig. 3). Analysis by STRING [11] identified interactants of wild-type MAL associated with post-Golgi vesicle-mediated protein transport, macromolecule localisation and protein localisation to membranes (Supplementary Fig. 4A). Pathway enrichment analysis using Database for Annotation, Visualisation and Integrated Discovery (DAVID v6.8) [12] identified plasma membrane components, intracellular vesicle-mediated transport, lipoprotein and membrane raft pathways (Supplementary Fig. 5A), consistent with the role of wild-type MAL as a lipoprotein involved in regulating PLP1 during trafficking from ER to membrane. Interactants of mutant MAL (Supplementary Fig. 4B) were enriched for pathways that included phagosome acidification and macroautophagy (Supplementary Fig. 5B), suggesting these proteins mediated ER stress or UPR as a result of MAL aggregates.

## Discussion

We present a missense variant in MAL as the likely cause of hypomyelinating leukodystrophy, resulting in ER mislocalization of mutant MAL leading to possible defects in PLP1 trafficking. One function of the ER is to assess the quality of newly-synthesised proteins, with misfolded proteins tending to form aggregates leading to ER stress [9]. ER stress then activates the UPR, in order to mitigate the stress by degrading the ER aggregates, diminishing protein translation and increasing the expression of ER chaperones [10, 13]. If the UPR fails, then cumulative ER stress will eventually lead to apoptosis [14]. The formation of ER aggregates caused by point mutations is a common disease mechanism for proteinopathies [15, 16], and is a possible pathomechanism for the MAL missense mutation p.(Ala109Asp). Our data suggests that p.(Ala109Asp) severely affects protein folding of MAL, leading to mislocalization in the ER. This was partly resolved by treatment with 4-phenylbutyrate, likely mediating correct folding of mutant MAL [17].

‘BioID’ proximity-dependent biotinylation identified potential protein interactions that provide insights into MAL function and how the p.(Ala109Asp) mutation affects these interactions. Interactions with wild-type MAL were grouped into cellular processes involving mainly vesicular transport. An example is VAPB, a protein mediating vesicle trafficking that is implicated in amyotrophic lateral sclerosis [18] and spinal muscular atrophy [19]. Another interactant was CKAP5, a cytoskeleton-associated protein, involved in the translation of myelin basic protein (MBP) consistent with a central role for MAL in myelin formation during neurodevelopment.

MAL acts as a regulator of PLP1 trafficking, redirecting PLP1 transport towards basolateral membranes during myelin formation [2]. In oligodendrocytes, MAL is not involved in PLP1 transport from the Golgi to the apical membrane, but it appears to redirect PLP1 trafficking from the apical membrane to the basolateral membrane where myelin begins to form [3]. Our data suggests that mutant p.(Ala109Asp) MAL, mislocalized in the ER, does not reach apical membranes where MAL and PLP1 interact. Loss of this interaction likely affects PLP1 transcytosis and subsequent myelin formation (Fig. 3C, E), thereby suggesting that the p.(Ala109Asp) missense variant is likely pathogenic on the basis of altered protein function.

In conclusion, this study describes the identification of a missense mutation in MAL that causes a neurodevelopmental condition characterised by hypomyelination and cerebellar atrophy. Our work supports a disease mechanism for leukodystrophies by which mislocalisation of MAL affects the distribution of PLP1 [20], resulting in a hypomyelination disorder similar to Pelizaeus-Merzbacher disease. This is consistent with the suggested pathomechanisms for hypomyelination that include defects in membrane integration or membrane interactions between proteins [20], for example transmembrane protein 106B (TMEM106B) that appears to mediate PLP1 trafficking.

## Supplementary information

Supplemental Material

Supplemental Figures

## Data Availability

The datasets generated and analysed during the current study are available from the corresponding author on reasonable request.
